# High Fat/High Glucose Diet Induces Metabolic Syndrome in an Experimental Rat Model

**DOI:** 10.3390/nu10101502

**Published:** 2018-10-14

**Authors:** Silvia Moreno-Fernández, Marta Garcés-Rimón, Gema Vera, Julien Astier, Jean François Landrier, Marta Miguel

**Affiliations:** 1 Bioactividady Análisis de los Alimentos, Instituto de Investigación en Ciencias de la Alimentación (CIAL, CSIC-UAM), Madrid 28049, Spain; silvia.moreno@csic.es; 2 Unidad Asociada I+D+i del Instituto de Investigación en Ciencias de la Alimentación (CIAL), Consejo Superior de Investigaciones Científicas (CSIC), Madrid 28049, Spain; gema.vera@urjc.es; 3 Grupo de investigación en Biotecnología Alimentaria, Instituto de Investigaciones Biosanitarias, Universidad Francisco de Vitoria, Pozuelo de Alarcón, Madrid 28233, Spain; marta.garces@ufv.es; 4 Departamento de Ciencias Básicas. Facultad de Ciencias de la Salud. Universidad Rey Juan Carlos, Alcorcón, Madrid 28922, Spain; 5 NORT, Aix-Marseille Université, INRA, INSERM, Marseille 13385, France; julien.astier@univ-amu.fr (J.A.); jean-francois.landrier@univ-amu.fr (J.F.L.)

**Keywords:** metabolic syndrome, obesity, diet induced obesity, rat model, high glucose, high fructose

## Abstract

Metabolic syndrome (MetS) is defined as a constellation of many metabolic disorders such as hypertension, impaired glucose tolerance, dyslipidemia and obesity, being this last disorder a key factor in the etiology of the syndrome. The widespread of MetS in actual society, mainly in developed countries, is becoming an important health problem and is increasing the need to develop new treatments against this pathology is increasing fast. The main objective of the present study was to evaluate the MetS-associated alterations developed in a new glucose diet-induced-obesity (DIO) rodent model. These alterations were also compared to those alterations developed in a fructose-DIO rodent model. Wistar rats were divided into four groups: Control (C), High-fat (HF), High-fat/high-fructose (HFF) and High-fat/high-glucose (HFG). The animals were fed ad libitum for 20 weeks. At the end of the study, HFG animals showed lower expression of energy expenditure genes when compared to the other DIO groups. Oxidative stress biomarkers such as MDA and mitochondrial RT-qPCR analyses showed an increase of oxidative damage together with mitochondrial dysfunction in HFG group. This group also showed increased insulin and glucose plasma levels, though HFF animals showed the greatest increase on these parameters. All DIO groups showed increased plasma levels of triglycerides. Altogether, our results indicated a better impact of glucose than fructose, when combined with a high-fat diet, to induce most of the alterations associated with MetS in rats. In addition, our research facilitates a new animal model to evaluate future treatments for MetS.

## 1. Introduction

Metabolic syndrome (MetS) is defined as a constellation of many metabolic disorders such as hypertension, impaired glucose tolerance, dyslipidemia and obesity, being this last disorder a key factor in the etiology of the syndrome [1,2]. MetS increases the incidence of cardiovascular diseases, type 2 diabetes (T2D) and nonalcoholic fatty liver disease [1,3,4]. The widespread of MetS in actual society, mainly in developed countries, is becoming an important health problem and the need to develop new treatments against this pathology is increasing fast.

To develop successful strategies to treat MetS, studies using animal models that adequately mimic all the aspects of human disease, developing all major alterations of the illness are needed. Regarding to this, as central obesity is a key factor in the development of MetS, Diet Induced Obesity (DIO) rodent models are frequently used to get better knowledge about the pathways implied in the development of MetS [1,3]. Currently, diets high in both carbohydrates and fat, are the most used models to resemble the nowadays human diet, also called “Western Diet” [3].

Actual society has experimented important changes in global food distribution and bioavailability. Changes in physical activities patterns together with the arrival of food processing centers producing energy-dense foods ready-to-eat, as well as the global presence of fast food establishments have carried to a westernized lifestyle in which sedentary and high fat-high carbohydrate dietary habits are alarmingly common [5,6]. Western diet is characterized by high caloric, energy-dense, take-away foods and sweetened beverages consumption. Carbohydrates present in this kind of diets are mainly sugars, being high-fructose corn syrup (HFCS) and sucrose the most commonly used sweeteners [5,7].

The increased use of HFCS and sucrose, together with the rising incidence of MetS in our society during last years, lead scientific community to postulate that high fructose consumption is related to the development of the pathology [1,8]. Fructose, commonly added as HFCS or sucrose in processed food, is believed to promote less satiety than other sugars, thus increasing caloric intake, mainly through sweetened beverages [8]; its different metabolism pathways compared with glucose one, suggests that fructose is the main cause in the development of MetS [1], and it is been traditionally used to develop DIO rodent models for MetS research [3,9,10].

However, it is important to note that sucrose, which contributes to more than 90% of caloric sweeteners globally, is a disaccharide containing equal amounts of fructose and glucose, while HFCS (main sweetener used in United States) differs little in its fructose and glucose contents (42–55% fructose: 45–58% glucose), and acts metabolically identical to sucrose [7]. Therefore, fructose and glucose intakes are similar in United States dietary patterns. In addition, due to HFCS (also called Isoglucose in Europe) production quota, HFCS is not as available in Europe as it is in United States, being more common the use of sucrose or products derived from glucose syrups, such as dextrose, a simple sugar made from corn, which is chemically identical to glucose and highly used in processed foods due to its versatility and its multiple food applications [11]. Even so, the prevalence of MetS in European society is increasing fast, as well as in United States. However, few animal models to investigate the influence of glucose or its derived products in MetS development have been carried out.

The aim of this study was to establish a new glucose-DIO rodent model and evaluate the MetS-associated alterations developed in the animals. In addition, this study compares the MetS-associated alterations developed in others previously stablished fructose-DIO rodent models. To do this, obesity was induced on Wistar rats by *ad libitum* intake of high-fat, high-fat/high-fructose or high-fat/high-glucose diets.

## 2. Materials and Methods

### 2.1. Experimental Protocol

The experiments were performed in strict accordance with the EC directive for the protection of animals used for scientific purposes (2010/63/UE) and the Spanish regulations (RD 53/2013), to minimize the number of animals used and their suffering. All experimental protocols were approved by the Ethical Committee of Universidad Rey Juan Carlos de Madrid (39/2012).

Wistar male rats at the age of 8 weeks (*n* = 29), weighting 280–310 g (Harlan Ibérica Barcelona, Spain) were housed at a constant temperature room (23 °C), with a 12 h light/dark cycles and free access to food and water.

After a week of adaptation, the animals were randomly assigned to four groups which received: standard chow diet (A04, SAFE, Augy, France) and tap water (Control group, C, *n* = 7), high-fat diet (Purified Diet 235 HF, SAFE, Augy, France) and tap water (High-fat diet group, HF, *n* = 6), high-fat diet and tap water with 25% fructose (High-fat/high-fructose diet group, HFF, *n* = 6) and high-fat diet and tap water with 25% glucose (High-fat/high-glucose diet group, HFG, *n* = 10), respectively, for twenty weeks. The solid and liquid diet compositions are detailed in Table 1. Throughout the feeding period, animals’ body weight was recorded weekly. Food and liquid intake were measured weekly and the data was used to calculate the energy intake of the different experimental groups. Over the experimental period, tactile allodynia (a neuropathic sign) was evaluated using the Von Frey hair test (see 2.2) at the beginning of the study and at week 6, 12 and 18.

At the end of the experimental period, rats were fasted for 16 h and then, the animals were anaesthetized with an intraperitoneal injection of ketamin (87 mg/kg) and xylazine (13 mg/kg), the abdominal circumference and body length (nose-to-anus length) were registered and used to estimate the body mass index (BMI). Finally, the rats were euthanized by decapitation. 

Blood was collected using lithium heparin tubes (BD Vacutainer, Oxford, UK) and plasma was obtained by blood centrifugation at 1500 g and 4 °C for 15 min, aliquoted and frozen at −80 °C until analysis. Heart, epididymal adipose tissue, kidneys, liver and salivary gland samples were excised, cleaned, weighed and frozen at −80 °C until analysis. Brown adipose tissue and soleus muscle were removed frozen at −80 °C until analysis. The length of the tibia was recorded.

### 2.2. Diabetic Neuropathy Evaluation: Von Frey Hair Test

Von Frey hair test was performed to evaluate the development of peripheral neuropathy following Vera et al. protocol [12]. A significant decrease in Von Frey hairs withdrawal threshold evoked by tactile-mechanical stimuli is suggestive of mechanical allodynia (increased sensitivity to non-noxious stimuli).

Over the experimental period, mechanical sensitivity was evaluated at the beginning of the study and at week 6, 12 and 18. Rats were put down individually on an elevated iron mesh and were allowed to adapt to new situation for at least 10 min. Adaptation to this environment was also performed on the day before assessment. Calibrated Von Frey hairs (4, 8, 10, 15, 26 and 60 g) were applied to the plantar aspect of each hind paw, from below the mesh floor. This test was repeated five times with 3 s intervals. Withdrawal responses to the stimulus were recorded and it was considered as positive result when at least three of five responses were obtained with each filament. This value was considered as the tactile threshold. When less than three positive responses were observed with any of the hair trials, the test was performed with the next higher force hair.

### 2.3. RNA Isolation and qPCR

Total RNA was obtained from tissues by TRIzol reagent (Life Technologies, Cergy Pontoise, France) according to the manufacturer’s manual. The cDNA was synthetized from 1 µg of total RNA using random primers and Moloney murine leukemia virus reverse transcriptase (Life Technologies) following the protocol described by Marcotorchino et al. [13]. Quantitative PCR analyses were carried out using the Mx3005P Real-Time PCR System (Stratagene, La Jolla, CA, USA). Expression was quantified in duplicate for each condition, and 18S rRNA was used as the endogenous control in the comparative cycle threshold (C_T_) method. Results were expressed as relative expression ratio [14]. The sequences of the primers used for qPCR analysis of gene expression are shown in Table S1.

### 2.4. Mitochondrial DNA Quantification

Total DNA was isolated from tissues using DNAzol (Euromedex, Strasbourg, France). The content of mitochondrial DNA (mtDNA) was determined using real-time quantitative PCR by measuring the threshold cycle ratio (ΔCT) of a mitochondrial-encoded gene Cox1 versus a nuclear-encoded gene cyclophilin A [15].

### 2.5. Oxidative Stress Biomarkers

*Antioxidant capacity of plasma*: The antioxidant capacity of plasma was evaluated by the oxygen radical absorbance capacity (ORAC) method, as previously described by Manso et al. [16], and measured fluorometrically (485 nm excitation and 520 emission; FLUOstar Optima, BMG Labtech GmbH, Offenburg, Germany). The results were expressed as µmol of trolox (Sigma, St. Louis, MO, USA) equivalent/µL of plasma.

*Plasma malondialdhehyde*: Plasma concentration of malondialdehyde (MDA) was determined by the thiobarbituric acid assay and spectrophotometrically measured at 535 nm (Infinite M200, Tecan, Switzerland) as previously reported by Manso et al. [16]. The results were expressed as nmol MDA/ml plasma.

*Glutathione Reduced in liver*: The levels of reduced glutathione (GSH) were measured by the monochlorobimane fluorimetric assay, previously reported by Kamencic et al., by a fluorometer (390 nm excitation and 510 emission; Infinite M200, Tecan, Switzerland) [17]. The results were expressed as µmol GSH/g protein.

### 2.6. Glucose Metabolism Determinations

Plasma concentrations of glucose were measured by a glucose-oxidase enzymatic commercial kit (Spinreact SAU, Sant Esteve de Bas, Spain) and determined at 540 nm with a spectrophotometer (Biotek HT Sinergy, BioTek Instruments, Inc., Winooski, VT, USA). Plasma levels of insulin were measured by an ultrasensitive insulin enzyme immunoassay commercial kit, specific for rat (Mercodia AB, Uppsala, Sweden) spectrophotometrically at 450 nm (Biotek HT Sinergy, BioTek Instruments, Inc., Winooski, VT, USA). Data were expressed as mg glucose and µmol insulin per milliliter of plasma, respectively.

Moreover, plasma concentrations of both glucose and insulin were used to calculate the insulin resistance index (homeostasis model assessment [HOMA]-IR) according to the following formula: fasting insulin (µU/mL) × fasting glucose (mM)/22.5 [18].

### 2.7. Lipid Metabolism/PLASMA Cholesterol and Triglycerides

Plasma concentrations of triglycerides and cholesterol were measured by enzymatic and colorimetric methods using commercial kits (Spinreact S.A/S.A.U, Sant Esteve de Bas, Spain), and spectrophotometrically determined at 450 nm (Biotek HT Sinergy, BioTek Instruments, Inc., Winooski, VT, USA). Results were expressed as mg of cholesterol and mg of triglycerides per milliliter of plasma, respectively.

### 2.8. Plasma Leptin and Adiponectin

Plasma concentrations of leptin and adiponectin were measured by ELISA kits (Cusabio, BioNova científica s.l., Madrid, Spain) specific for rat. The results were expressed as ng leptin and µg adiponectin per milliliter of plasma, respectively.

### 2.9. Statistical Analysis

Results are expressed as mean values ± S.E.M. for a minimum of 6 animals. For statistical analysis, Student t test and one-way analysis of variance (ANOVA) were used for parametric results; differences between the groups were evaluated by the Bonferroni post-hoc test, using GraphPad Prism 6 software (Graph Pad, San Diego, CA, USA). Non-parametric results were analyzed using Mann-Whitney test. *p* < 0.05 was considered to be statistically significant.

## 3. Results

### 3.1. High-Fat/High-Glucose Diet Induces a Greater Weight Gain and Lower Energy Expenditure

**Figure 1:**
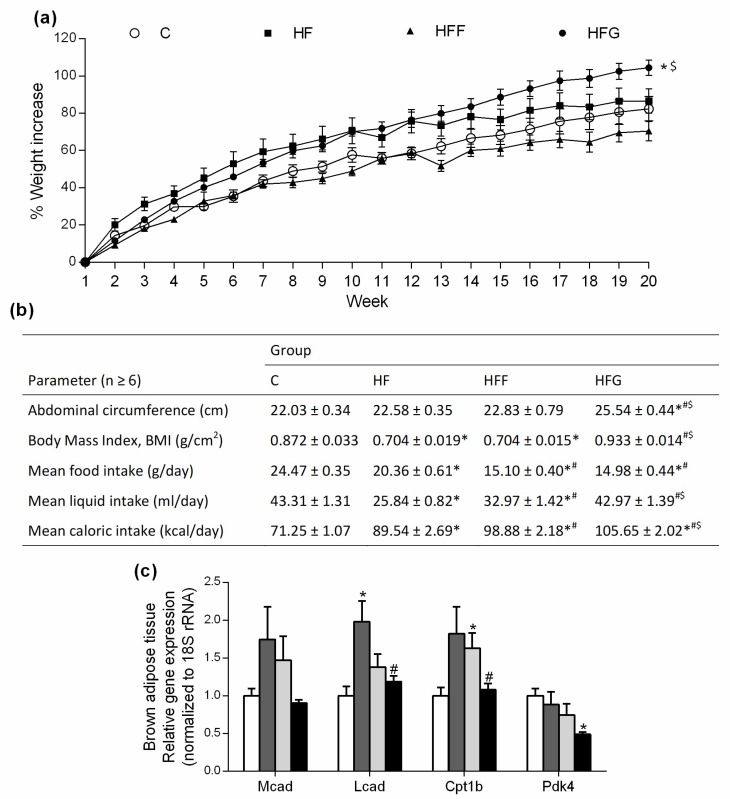
Body weight, anthropometrics and energy metabolism results: (**a**) Body weight gain during the study; (**b**) Anthropometric results at the end of the study, (**c**) relative expression in brown adipose tissue of genes related to energy expenditure. Experimental groups: Control group (C), High-fat diet group (HF), High-fat/high-fructose diet group (HFF) and High-fat/high-glucose diet group (HFG). Values are means ± SEM (*n* ≥ 6). * *p* < 0.05 vs C; # *p* < 0.05 vs HF; $ *p* < 0.05 vs HFF.

Body weight was significantly higher in HFG animals at the end of the study (week 20th) compared to C and HFF groups (Figure 1a), no differences were found when compared HFG animals vs HF animals. HFF rats did not increase their body weight, compared to control group. As shown in Figure 1b, HFG was the only DIO group that significantly increased its abdominal circumference, and no significant changes were observed in HF or HFF group when compared to C group. Moreover, HF and HFF animals significantly decreased their BMI, compared with C group, while HFG animals did not experienced a significant change in this parameter. All DIO groups showed a significant decrease in food intake when compared the registered data to C group; this decrease was specially marked in sugar-fed groups (HFF and HFG). Regarding liquid intake, during all experimental period HF and HFF groups significantly decreased their liquid intake when compared to C animals, while HFG kept this parameter unchanged respecting controls. Considering caloric intake, it was observed significant differences between all groups, and HFG animals showed the highest caloric intake comparing all the groups. Energy expenditure biomarkers in brown adipose tissue were also measured (Figure 1c). While Medium-Chain Acyl-CoA Dehydrogenase (Mcad) stayed unchanged within all experimental groups, Long-Chain Acyl-CoA Dehydrogenase (Lcad) was significantly more expressed in HF animals when compared to C group; Carnitine palmitoyltransferase 1β (Cpt1B) was significantly more expressed in HFF animals compared to C animals. The expression of both Lcad and Cpt1B did not show significant changes in HFG group when compared to controls. Pyruvate dehydrogenase kinase 4 (Pdk4) was significantly less expressed in HFG compared to C animals; no significant differences were observed in HF or HFF groups when compared to C or HFG animals.

As shown in Table 2, relative weight of epididymal adipose tissue was significantly doubled in HFG group when compared to C animals. This parameter was also increased in HF animals when compared to C, while stayed unchanged in HFF animals. Liver relative weight did not show significant changes in any DIO groups compared to controls.

### 3.2. High-Fat/High-Glucose Diet Increases Lipid Peroxidation and Induces Mitochondrial Dysfunction in Brown Adipose Tissue

**Figure 2:**
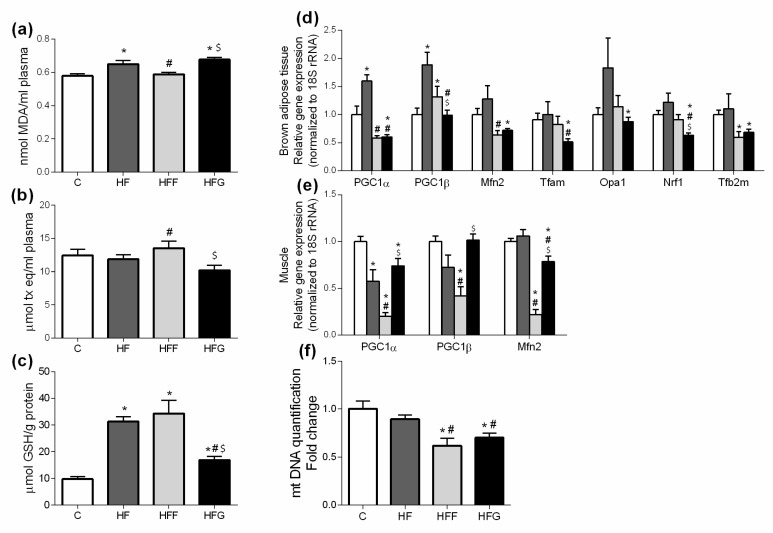
Oxidative stress biomarkers: (**a**) Plasma malondialdehyde (MDA) levels; (**b**) Plasma antioxidant capacity; (**c**) Liver reduced glutathione (GSH) levels; (**d**) Gene expression in brown adipose tissue and (**e**) muscle of genes implied in mitochondrial dynamics regulation; (**f**) Mitochondrial DNA (mtDNA) quantification in brown adipose tissue. Experimental groups: Control group (C), High-fat diet group (HF), High-fat/high-fructose diet group (HFF) and High-fat/high-glucose diet group (HFG). Values are means ± SEM (*n* ≥ 6). * *p* < 0.05 vs C; # *p* < 0.05 vs HF; $ *p* < 0.05 vs HFF.

Plasma levels of MDA were significantly increased in HF and HFG, while remained unchanged in HFF group (Figure 2a). When compared all DIO groups to C animals, no significant changes in plasma antioxidant capacity were observed (Figure 2b). However, HFF group showed a significant increase in this parameter compared to HF and HFG animals. Regarding to GSH levels in liver (Figure 2c), all studied DIO groups significantly increased this parameter compared to control animals, being significantly higher in HF and HFF groups compared to HFG animals.

Figure 2d,e show expression of genes related to mitochondrial biogenesis and dynamics in brown adipose tissue and muscle. As shown in the figure, most of the measured genes related to mitochondrial biogenesis and dynamics (PPARγ Coactivator 1α, PGC1α; Mitofusin-2, Mfn2; Mitochondrial transcription factor A, Tfam; Mitochondrial dynamin-like GTPase, Opa1; Nuclear respiratory factor 1, Nrf1; Mitochondrial Transcription Factor B2, Tfb2m) in brown adipose tissue were significantly under expressed in HFG rats, compared to control group; PGC1α and PPARγ Coactivator 1β (PGC1β) were significantly more expressed in brown adipose tissue of HF rats compared to controls; HFF rats significantly increased PGC1β expression in brown adipose tissue, and significantly lower expression of Mfn2 and Tfb2m, compared to C group.

Expression of most of the analyzed genes related to mitochondrial dynamics in muscle were significantly lower in all DIO groups compared to controls (Figure 2e); however, this decrease was significantly more remarkable in HFF rats, compared to HF and HFG animals.

Regarding to mtDNA content in brown adipose tissue (Figure 2f), both HFF and HFG decreased this parameter when compared to control animals. No differences were found in HF group when compared to C animals.

### 3.3. High-Fat/High-Glucose Diet Increases Fasting Glucose and Insulin Levels in Plasma

**Figure 3:**
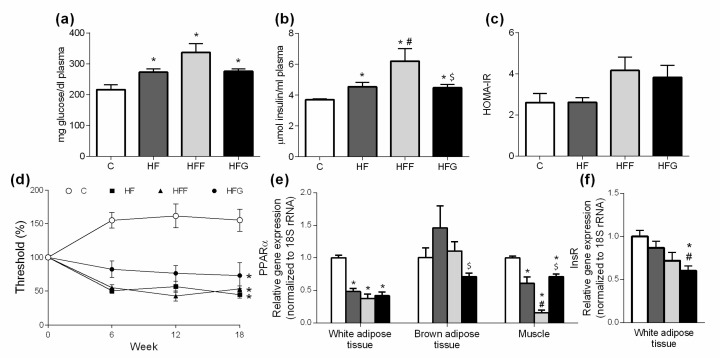
Glucose metabolism biomarkers: (**a**) Plasma glucose levels; (**b**) Plasma insulin levels; (**c**) HOMA-IR calculated at the end of the study; (**d**) Mechanical sensitivity evolution during the study; (**e**) Gene expression in white adipose tissue, brown adipose tissue and muscle of PPARα; (**f**) gene expression in white adipose tissue of insulin receptor (InsR). Experimental groups: Control group (C), High-fat diet group (HF), High-fat/high-fructose diet group (HFF) and High-fat/high-glucose diet group (HFG). Values are means ± SEM (*n* ≥ 6). * *p* < 0.05 vs C; # *p* < 0.05 vs HF; $ *p* < 0.05 vs HFF.

As shown in Figure 3a, at the end of the study, all DIO groups significantly increased fasting glucose levels in plasma when compared to control group. Plasma levels of insulin (Figure 3b) were also increased in DIO animals compared to controls and insulin levels were significantly higher in HFF group compared to HF and HFG groups. Changes observed in glucose and insulin levels are however not represented in HOMA-IR index, since significant differences between groups were not found in this parameter (Figure 3c). Evaluation of tactile allodynia manifestation (Figure 3d) showed that all DIO groups significantly reduced their mechanical threshold, being these differences significant since week 6th, compared to C group. Gene expression of Peroxisome proliferator activated receptor α (PPARα) in epididymal adipose tissue (Figure 3e) was significantly lower in all DIO groups compared to C animals. Insulin receptor (InsR) expression in this tissue was significantly lower only in HFG group when compared to C group. PPARα expression in brown adipose tissue was slightly reduced in HFG animals, although significant differences were found just when compared to HFF animals. Regarding to PPARα gene expression in muscle, all DIO showed a significant decrease in this parameter when compared to C group. This decrease was significantly important in HFF animals when compared to HF and HFG groups.

### 3.4. High-Fat/High-Glucose Diet Increases Plasma TG Levels and Lipids Mobilization in White Adipose Tissue

**Figure 4:**
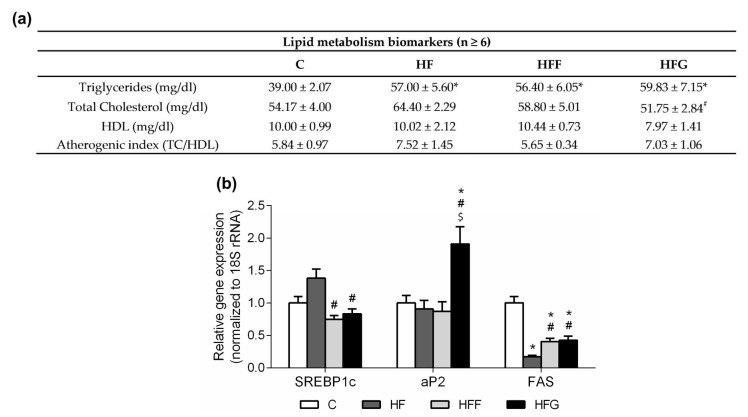
Results in lipid metabolism biomarkers. (**a**) Results in plasma lipid metabolism biomarkers at the end of the study; (**b**) expression of genes related with lipid metabolism in epididymal adipose tissue. Experimental groups: Control group (C), High-fat diet group (HF), High-fat/high-fructose diet group (HFF) and High-fat/high-glucose diet group (HFG). Values are means ± SEM (*n* ≥ 6). * *p* < 0.05 HF, HFF, HFG vs C; # *p* < 0.05 HFF, HFG vs HF; $ *p* < 0.05 HFG vs HFF.

Plasma TG levels were significantly increased in all DIO groups compared to C group, as shown in Figure 4a, no significant differences between DIO groups were observed in this parameter. No differences were found in plasma total cholesterol levels when DIO animals were compared to C animals, however HF group showed this parameter significantly increased when compared to HFF animals, while HFG animals showed this parameter significantly decreased when compared to HFF group. Regarding plasma HDL levels, no differences between groups were observed although HFG showed a slight decrease of this parameter. Atherogenic index was slightly increased in HF and HFG animals, although no significant differences were found between groups.

Regarding expression of genes related to lipid metabolism and mobilization in epididymal adipose tissue (Figure 4b), no significant differences were observed in Sterol Regulatory Element-Binding Protein 1c (SREBP1c) gene expression in any DIO group when compared to C animals. HF animals showed an increased expression of SREBP1c when compared to HFF and HFG animals. No differences were observed between HFF and HFG animals in this parameter. Gene expression of Adipocyte Protein 2 (aP2) was significantly raised in HFG animals when compared to the other experimental groups. Fatty Acid Synthase (FAS) was less expressed in all DIO animals when compared to controls, being this reduction less pronounced in HFF and HFG groups compared to HF group.

### 3.5. High-Fat/High-Glucose Diet Could Induce Adiponectin Resistance

**Figure 5:**
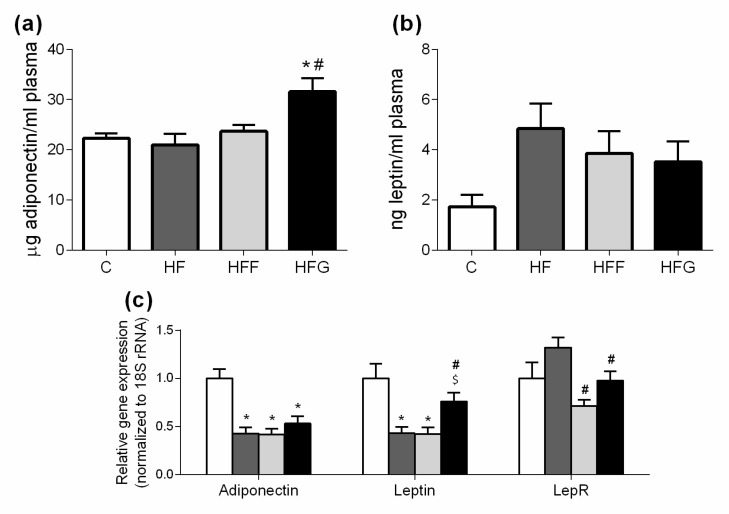
Plasma levels of adiponectin and leptin at the end of the study: (**a**) Plasma adiponectin levels; (**b**) plasma leptin levels; (**c**) Gene expression of adiponectin, leptin and leptin receptor (LepR) in white adipose tissue. Experimental groups: Control group (C), High-fat diet group (HF), High-fat/high-fructose diet group (HFF) and High-fat/high-glucose diet group (HFG). Values are means ± SEM (*n* ≥ 6). * *p* < 0.05 vs C; # *p* < 0.05 vs HF; $ *p* < 0.05 vs HFF.

As shown in Figure 5a, plasma adiponectin levels were significantly increased in HFG group compared to C animals, while HF and HFF keep this parameter unchanged. Plasma leptin levels did not significantly change in any DIO group compared to control animals (Figure 5b).

Figure 5c shows a reduction of adiponectin gene expression in epididymal adipose tissue of all DIO animals, compared to controls. Both HF and HFF groups showed a significantly lower expression of leptin than C group, while HFG did not change this parameter. HF animals significantly increased the expression of LepR gene when compared to HFF and HFG animals, but this difference was not significant when compared to C group.

## 4. Discussion

The rising incidence of MetS and the increased consumption of fructose and sucrose in western societies during last years, lead scientific community to postulate that at least, high fructose consumption is related to the development of this pathology [1,8]. However, considering that fructose and glucose intakes are similar in USA dietary patterns, and glucose intake is even higher in Europe, there are not studies so far in which the influence of glucose during MetS development is proved.

The primary focus of this study was to characterize physical and metabolic changes produced due to a high-fat/high-glucose diet consumption, compared to animals fed a standard chow diet. In addition, a secondary focus of this study was to compare if these changes were as different as expected when compared to those developed with a high-fat or high-fat/high-fructose diet intake.

In our study, animals fed a high-fat/high-glucose diet showed an increase in body weight, fat deposition, an increase in oxidative stress biomarkers, raised fasting levels of glucose and insulin in plasma and allodynia (a feature of neuropathic pain) which suggest an early stage MetS development. These alterations were in addition more marked in glucose-fed animals when compared to fructose-fed ones, showing clear differences depending on sugar consumption and the key role of glucose on MetS development.

Weight gain is caused by an imbalance between energy uptake and energy expenditure that causes fat accumulation in white adipose tissue [19]. In our study, all DIO groups showed an increase in caloric intake, but just HFG animals showed an increase in body weight. It is important to note that, although no significant differences were found against controls, HFF animals presented the lowest body weight at the end of the study. Despite several studies have proved that high-fat/high-fructose diets induce an increase in body weight, abdominal fat deposition, abdominal circumference and BMI [1,3,9], we did not obtain those results in our study. It is possible that, to induce such fat deposition and weight gain, it could be necessary to feed a higher amount of fructose to the animals, reaching a 40–70% of energy from fructose in the diets [1,9]. This amount of fructose might not be a realistic intake of sugars, being glucose a better choice to induce fat deposition and body weight gain by mimicking a real human diet. In addition, we observed that both HFF and HFG groups reduced their food intake compared to controls. HFF animals reduced their liquid intake as well, while HFG animals did not change this parameter when compared to controls. Although it has been observed that fructose is less able to promote satiety, thus increasing caloric intake [8], our results showed a reduced caloric intake in HFF animals. As fructose is around twice as sweet as glucose [11], the reduced caloric intake seems to be caused by a reduced liquid intake due to a sensory-specific satiety. Sensory-specific satiety is defined as the declining pleasure and attraction to the sensory attributes to a specific food eaten in a meal [20]. Regarding to this, a glucose solution could be more pleasant to drink than a fructose solution, thus reducing the sweet-related satiety, resulting in a major consumption of glucose under same conditions. Our results imply that, when we work on an animal model under *ad libitum* situations, the utilization of glucose as sugar makes able to double caloric intake respecting models in which fructose is used, thus accelerating the development of metabolic damages and speeding up the induction of MetS.

Among the huge variety of scales available to measure obesity, BMI and abdominal circumference are two of the most common scalesused to predict the development of obesity-associated pathologies [19]. Although these measures are to some extent influenced by age, gender and ethnicity, and despite the controversial use of BMI to predict metabolic risk, both high BMI and/or abdominal circumference are still considered as valuable markers for predicting metabolic disorders and related diseases [19,21,22]. Abdominal circumference reflects indeed visceral adipose tissue, which has a stronger relationship with MetS due to its important secretion of pro-inflammatory adipokines [23]. Regarding to this, HF and HFF groups did not show an increase in abdominal circumference compared to C animals, but they even showed a lower BMI. Both combined results are related with a reduced MetS risk factor. On the other hand, HFG showed a significant increase in abdominal circumference, which occurs together with an increase in epididymal adipose tissue weight. Overall, anthropometrics results suggest a major risk to develop MetS in HFG rats compared to the other DIO animals [19,21,22].

Due to all the animals in the present study were sedentary, thermogenesis became the main way to expend the excess of caloric intake due to a high-fat/high-sugar diet. Lcad and Cpt1B, both implicated in fatty acid oxidation, are highly expressed in HF and HFF animals, which lead to a higher energy expenditure from fatty acids, while expression of these genes remain unchanged in HFG. On the other hand, Pdk4 is downregulated in HFG, which could lead to an activation of pyruvate dehydrogenase and a higher expenditure of energy provided from glucose in this tissue. It has been already observed that DIO Wistar rats exhibited an upregulation of brown-fat markers in white adipose tissue as an adaptive mechanism against a high caloric diet. Brown fat depots in white adipose tissue increase fatty acid and sugar oxidation leading to a lower fat accumulation in adipocytes [24,25]. Our results suggest that fatty acid and sugar oxidation stimulation also occur in brown adipose tissue. However, upregulation of fatty acid oxidation seems to be more effective than upregulation of glucose oxidation in order to burn energy, as we observed an increased adiposity and body weight gain in HFG in comparison to HF and HFF animals.

Oxidative stress, mainly caused by mitochondrial dysfunction, is strongly linked with MetS development. Excessive energy supply leads to an increased oxidative activity that, along with an insufficient antioxidant defense, causes an overproduction of reactive oxygen species (ROS) in mitochondria, causing damages in other macromolecules such as lipids, proteins and nucleic acids [4,26,27]. Malondialdehyde (MDA) plasma levels were increased in HF and HFG groups, while stay unchanged in HFF animals. HFF group even presented higher plasma antioxidant capacity than the other DIO animals. These results suggest a better capability of glucose than fructose to induce oxidative damage when combined with a high fat diet. An excessive generation of free radicals in the cells induces the activation of defense mechanisms, such as antioxidant enzymes which protect the cells against oxidative stress. In our study, we found an overproduction of liver GSH in all DIO animals, which could confirm the activation of the antioxidant defense. Shawky et al. did already observe this GSH overproduction in rats [28]. However, GSH levels in HFG were significantly lower than in HFF or HF livers, probably due to a greater production of ROS underlying in HFG group. Besides, genes related to mitochondrial biogenesis and dynamics (PPARα, PGC1α, Mfn2, Tfam, Opa1, Nrf1, Tfb2m) were downregulated in brown adipose tissue of HFG animals when compared to C. This general downregulation is less pronounced in HFF, showing even an upregulation of PGC1β compared to C. Mitochondrial biogenesis and dynamics are very important to keep mitochondrial machinery and genome [4], being the downregulation of these dynamics in HFG a strong indicator of mitochondrial damage and dysfunction.

Both abdominal adiposity and oxidative stress are strongly related to insulin resistance, impaired glucose metabolism and, eventually, the development of Type 2 Diabetes Mellitus (T2DM) [1,29]. Regarding this, it is important to note that both glucose and insulin plasma levels were increased in all DIO groups, being this change stronger in HFF. However, HOMA-IR did not show significant differences, although an increasing trend was observed in both HFF and HFG groups. Regarding the role of PPARα controlling fatty acids oxidation, its downregulation has been related with insulin resistance [30]. As expected, PPARα was strongly downregulated in white adipose tissue and muscle of all DIO animals. However, just HFG animals showed a significant downregulation of insulin receptor (InsR) in white adipose tissue when compared to C.This result could be related with the slight reduction in PPARα expression observed in brown adipose tissue of HFG animals. Sensory neuropathy occurrence has been related with insulin resistance and appears at early stages of diabetes, even before changes on insulin or glucose plasma levels can be noticed [31]. In our study, measurement of tactile allodynia confirmed that all the DIO animals developed sensory neuropathy. High-fructose diets have been widely used to develop insulin-resistance animal models either combined or not with high-fat diets [32]. However, these kind of models usually require high content of fructose in the diets (50–70%), large periods of high-fructose feeding or even the utilization of old-animals in order to develop metabolic alterations [32,33,34]. In our study, young rats were fed a low-sugar-content solution (25%) during 20 weeks, which would not be enough to induce metabolic damage in the animals. Our results suggest that fructose could be better to induce impaired glucose metabolism than glucose itself, when combined to a high-fat diet. However, even though all the alterations measured were more marked in HFF animals, it is important to highlight the capability of glucose to induce them, being possible the development of insulin resistance and impaired glucose tolerance under a higher content of glucose fed to the animals or using older animals in the study.

When consumed, fructose is rapidly absorbed by liver, transformed into fatty acids and then released to systemic circulation in form of TG thus increasing TG plasma levels and atherogenic risk [1,8]. In our study, all DIO animals, even HF group, showed an increase in TG levels, and they did not show changes in total cholesterol nor HDL levels when compared to controls. Regarding this, we cannot attribute the increase of the TG levels to the intake of sugar, but to the intake of fat itself. In this way, both glucose and fructose could just promote a stronger change in this parameter. During obesity, excess energy is stored in white adipose tissue in form of fatty acids and TG, leading to adipocytes hypertrophy [23,35]. When adipocytes are overloaded, excess of fatty acids are released back to systemic circulation and ectopic fat accumulation occurs, aggravating MetS complications [35]. SREBP1c acts as the main regulator of lipogenesis in white adipose tissue. This gene showed to be overexpressed in HF group when compared with HFF and HFG, although no differences were observed when the DIO groups where compared to control animals. These results suggested a stimulated lipogenesis en HF animals when compared to the groups which consumed sugar during the feeding period (HFF and HFG groups). In addition, FAS expression was reduced in all DIO animals, this is an expected result, as there is an excessive supply of fatty acids result of a high-fat diet intake. However, both HFF and HFG groups showed a higher expression of FAS when compared to HF animals. This is probably due to an excess of circulating sugar, leading to a stimulation of fatty acids and TG synthesis. On the other hand, aP2 only increased its expression in HFG animals, suggesting that just this group experienced an important mobilization of fatty acids in adipocytes, and this is the only group in which adipocytes are overloaded.

Regarding the implication of inflammatory response in the development of MetS, our results showed an unexpected increase in adiponectin plasma levels in HFG group when compared to control, in spite of a reduced gene expression of this anti-inflammatory adipokine in white adipose tissue of all DIO groups. This controversial result, already observed by other researchers [36,37], seems to occur at early stages of metabolic syndrome development [37] or during weight gain events [38]. Certainly, some researchers have observed that the impairment of adiponectin secretion and the lowering of adiponectin levels in plasma arise just after metabolic syndrome establishment [37]. Although the mechanisms involved in this event are still unclear, adiponectin resistance has been proposed as a possible cause, leading to the accumulation of this adipokine in the systemic circulation [37,39]. Actually, Tsuchida et al. observed a lower expression of adiponectin receptors during hyperinsulinemic and hyperglycemic states [39]. On the other hand, leptin plasma levels showed an increasing trend although no significant differences were observed. As a resistance mechanism was expected, we measured gene expression of both genes in white adipose tissue: Leptin protein itself and LepR, so that we can observe an increase on leptin sensitivity in HF animals, due to an increase in LepR expression. Although significant decreasing was not observed in LepR expression nor in HFF nor HFG, we could observe a significant decrease of leptin gene expression in HF and HFF animals, while HFG did not change its expression when compared to control. These results in leptin and adiponectin levels, suggest a low-grade inflammatory response in HFG, stronger than in HFF animals. 

Several researchers have already disputed claims about the importance of fructose in MetS development over other kind of sugars, such as glucose [7,40]. In this way, Rippe and Angelopoulos insist that, given the complexity of weight gain and energy regulation, it is unlikely that just one component of the diet impacts upon the problem, and claims that greater intake of sugars lead to overconsumption of calories leading to weight gain [40]. However, our results have shown that there are indeed differences between type of sugar consumption, glucose or fructose, in MetS development. Compared to fructose, glucose induces a major adiposity and oxidative stress status, which accelerate metabolic abnormalities and MetS manifestation. It is important to note that, due to HFCS production quota, this source of fructose is not as available in Europe as it is in United States, being glucose syrups and derived products commonly used in Europe [11].

In brief, High-fat/high-glucose fed rats showed an increased body weight, abdominal circumference and fat deposition, combined with oxidative stress, raised fasting levels of glucose and insulin in plasma, dyslipidemia and mechanical allodynia. Altogether, our results showed an early-stage MetS which, in addition, could lead to T2DM and cardiovascular disease development over time in HFG animals. This study suggests that we could be paying too much attention to fructose, leading us to overlook the important contribution of glucose (also called dextrose in food industry) in MetS development. Our study demonstrates the effectivity of glucose over fructose to induce MetS in animals when it is combined with a high-fat diet. However, more studies are necessary in order to characterize in deep the high-fat/high-glucose model. These studies should consider the limitations of this study and to include data for classical glucose and insulin tolerance tests and also to explore the cardiovascular condition of this new experimental model, since MetS is an important risk factor for cardiovascular disease. In conclusion, the high-fat/high-glucose model could be useful to study novel therapeutic agents for controlling MetS and related complications.

## 5. Conclusions

In brief, High-fat/high-glucose fed rats showed an increased body weight, abdominal circumference and fat deposition, combined with oxidative stress, impaired glucose tolerance, dyslipidemia and mechanical allodynia. Altogether, our results showed an early-stage MetS which, in addition, could lead to T2DM and cardiovascular disease development over time in HFG animals. This study suggests that we could be paying too much attention to fructose, leading us to overlook the important contribution of glucose (also called dextrose in food industry) in MetS development. Our study demonstrates the effectivity of glucose over fructose to induce MetS in animals when it is combined with a high-fat diet. However, more studies are necessary in order to characterize more deeply the high-fat/high-glucose model and also to investigate the cardiovascular condition of this new experimental model, since MetS is an important risk factor for cardiovascular disease. In conclusion, the high-fat/high-glucose model could be useful to investigate novel therapeutic agents for controlling MetS and related complications.

## Figures and Tables

**Table 1:** Composition of the solid and liquid diets administered during the study to the different groups.

Composition	Basal Diets		Sugar Solutions	
	Chow Diet	High Fat Diet	25% Dextrose Solution	25% Fructose Solution
Carbohydrate, %	60	42.3	25	25
of which sugars, %	-	-	25	25
Protein, %	16	17	-	-
Fat, %	3	22.5	-	-
Fiber, %	4	3.2	-	-
Minerals, %	5	5	-	-
Moisture, %	12	10	75	75
Energy density, kcal/g, kcal/mL	2.900	4.397	0.913	1.000

**Table 2:** Tissue and organ relative weights at the end of the study.

Organ (g/cm Tibial Length) (*n* ≥ 6)	Group			
	C	HF	HFF	HFG
Epididymal adipose tissue	3.70 ± 0.24	4.94 ± 0.27 *	3.98 ± 0.53	6.28 ± 0.52 *^$^
Liver	2.90 ± 0.09	3.01 ± 0.08	3.12 ± 0.10	3.15 ± 0.10

Experimental groups: Control group (C), High-fat diet group (HF), High-fat/high-fructose diet group (HFF) and High-fat/high-glucose diet group (HFG). Values are means ± SEM (*n* ≥ 6). * *p* < 0.05 vs C; $ *p* < 0.05 HFF.

## Supplementary Materials

The following are available online at http://www.mdpi.com/2072-6643/10/10/1502/s1, Table S1: Primers used for RT-qPCR in the study.

## References

[1] Aydin S., Aksoy A., Aydin S., Kalayci M., Yilmaz M., Kuloglu T., Citil C., Catak Z. (2014). Today’s and yesterday’s of pathophysiology: Biochemistry of metabolic syndrome and animal models. Nutrition.

[2] Merone L., McDermott R. (2017). Nutritional anti-inflammatories in the treatment and prevention of type 2 diabetes mellitus and the metabolic syndrome. Diabetes Res. Clin. Pract..

[3] Panchal S., Brown L. (2011). Rodent models for metabolic syndrome research. J. Biomed. Biotechnol..

[4] Bhatti J.S., Bhatti G.K., Reddy P.H. (2017). Mitochondrial dysfunction and oxidative stress in metabolic disorders–A step towards mitochondria based therapeutic strategies. Biochim. Biophys. Acta.

[5] Assifi M.M., Eibl G., Shankar S., Srivastava R.K. (2012). Western diet-induced pancreatic cancer. Nutrition, Diet and Cancer.

[6] Popkin B.M. (2015). Nutrition transition and the global diabetes epidemic. Curr. Diab. Rep..

[7] Johnston R.D., Stephenson M.C., Crossland H., Cordon S.M., Palcidi E., Cox E.F., Taylor M.A., Aithal G.P., Macdonald I.A. (2013). No difference between high-fructose and high-glucose diets on liver triacylglycerol or biochemistry in healthy overweight men. Gastorenterology.

[8] Martins Pereira R., Botezelli J.D., da Cruz Rodriges K.C., Mekary R.A., Cintra D.E., Pauli J.R., da Silva A.S.R., Ropelle E.R., de Moura L.P. (2017). Fructose consumption in the development of obesity and effects of different protocols of physical exercise on the hepatic metabolism. Nutrients.

[9] Panchal S.K., Poudyal G., Iyer A., Nazer R., Alam A., Diwan V., Kauter K., Sernia C., Campbell F., Ward L. (2011). High-carbohydrate, high-fat diet-induced metabolic syndrome and cardiovascular remodeling in rats. J. Cardiovasc. Pharmacol..

[10] Crescenzo R., Bianco F., Coppola P., Mazzoli A., Cigliano L., Liverini G., Iossa S. (2015). The effect of high-fat-high-fructose diet on skeletal muscle mitochondrial energetics in adult rats. Eur. J. Nutr..

[11] Hull P. (2010). Glucose Syrups: Technology and Applications.

[12] Vera G., Chiarlone A., Cabezos P.A., Pascual D., Martín M.I., Abalo R. (2007). WIN 55,212-2 prevents mechanical allodynia but not alterations in feeding behaviour induced by chronic cisplatin in the rat. Life Sci..

[13] Marcotorchino J., Tourniaire F., Astier J., Karkeni E., Canault M., Amiot M.J., Bendahan D., Berdard M., Martin J.C., Giannesini B. (2014). Vitamin D protects against diet-induced obesity by enhancing fatty acid oxidation. J. Nutr. Biochem..

[14] Livak K.J., Schmittgen T.D. (2001). Analysis of relative gene expression data using real-time quantitative PCR and the 2-ΔΔCt method. Methods.

[15] Tourniaire F., Musinovic H., Gouranton E., Astier J., Marcotorchino J., Arrequin A., Bernot D., Palou A., Bonet M.L., Ribot J. (2015). All-trans retinoic acid induces oxidative phosphorylation and mitochondrial biogenesis in adipocytes. J. Lipid Res..

[16] Manso M.A., Miguel M., Even J., Hernández J., Aleixandre A., López-Fandiño R. (2008). Effect of the long-term intake of an egg white hydrolysate on the oxidative status and blood lipid profile of spontaneously hypertensive rats. Food Chem..

[17] Kamencic H., Lyon A., Paterson P.G., Juurlink B.H.J. (2000). Monochlorobimane fluorometric method to measure tissue glutathione. Anal. Biochem..

[18] Fenni S., Hammou H., Astier J., Bonnet L., Karkeni E., Couturier C., Tourniaire F., Landrier J.F. (2017). Lycopene and tomato powder supplementation similarly inhibit high-fat diet induced obesity, inflammatory response, and associated metabolic disorders. Mol. Nutr. Food Res..

[19] Seo D.C., Choe S., Torabi M.R. (2017). Is waist circumference ≥ 102/88 cm better than body mass index ≥ 30 to predict hypertension and diabetes development regardless of gender, age group, and race/ethnicity? Meta-analysis. Prev. Med..

[20] Myers K.P. (2017). Sensory-specific satiety is intact in rats made obese on a high-fat high-sugar choice diet. Appetite.

[21] Vasques A.C.J., Cassani R.S.L., Forti A.C., Vilela B.S., Pareja J.C., Tambascia M.A., Geloneze B., BRAMS investigators (2015). Sagittal abdominal diameter as a surrogate marker of insulin resistance in an admixtured population—Brazilian Metabolic Syndrome Study (BRAMS). PLoS ONE.

[22] Delvarianzadeh M., Abbasian M., Khosravi F., Ebrahimi H., Ebrahimi M.H., Fazli M. (2017). Appropriate anthropometric indices of obesity and overweight for diagnosis of metabolic syndrome and its relationship with oxidative stress. Diabetes Metab. Syndr..

[23] Schrover I.M., Spiering W., Leiner T., Visseren F.L. (2016). Adipose tissue dysfunction: clinical relevance and diagnostic possibilities. Horm. Metab. Res..

[24] García-Ruiz E., Reynés B., Díaz-Rúa R., Ceresi E., Oliver P., Palou A. (2015). The intake of high-fat diets induces the acquisition of brown adipocyte gene expression features in white adipose tissue. Int. J. Obes..

[25] Trayhurn P. (2017). Origins and early development of the concept that brown adipose tissue thermogenesis is linked to energy balance and obesity. Biochimie.

[26] Bonomini F., Rodella L.F., Rezzani R. (2015). Metabolic syndrome, aging and involvement of oxidative stress. Aging Dis..

[27] Boulinguiez A., Staels B., Duez H., Lancel S. (2017). Mitochondria and endoplasmic reticulum: Targets for a better insulin sensitivity in skeletal muscle?. Biochim. Biophys. Acta Mol. Cell Biol. Lipids.

[28] Shawky N.M., Hehatou G.S.G., Rahim M.A., Suddek G.M., Gameil N.M. (2014). Levocetirizine ameliorates high fructose diet-induced insulin resistance, vascular dysfunction and hepatic steatosis in rats. Eur. J. Pharmacol..

[29] Hafizi Abu Bakar M., Kian Kai C., Wan Hassan W.N., Sarmidi M.R., Yaakob H., Zaman Huri H. (2015). Mitochondrial dysfunction as a central event for mechanisms underlying insulin resistance: The roles of long chain fatty acids. Diabetes Metab. Res. Rev..

[30] Rakhshandehroo M., Knoch B., Müller M., Kersten S. (2010). Peroxisome proliferator-activated receptor alpha target genes. PPAR Res..

[31] Lupachyk S., Watcho P., Hasanova N., Julius U., Obrosova I.G. (2012). Triglyceride, nonesterified fatty acids, and prediabetic neuropathy: Role for oxidative-nitrosative stress. Free Radic. Biol. Med..

[32] Sah S.P., Singh B., Choudhary S., Kumar A. (2016). Animal models of insulin resistance: A review. Pharmacol. Rep..

[33] Maithlikarpagaselvi N., Sridhar M.G., Swaminathan R.P., Zachariah B. (2016). Curcumin prevents inflammatory response, oxidative stress and insulin resistance in high fructose fed male Wistar rats: Potential role of serine kinases. Chem. Biol. Interact..

[34] Putakala M., Gujjala S., Nukala S., Desireddy S. (2017). Beneficial effects of Phyllanthus amarus against high fructose diet induced insulin resistance and hepatic oxidative stress in male wistar rats. Appl. Biochem. Biotechnol..

[35] Grundy S.M. (2016). Overnutrition, ectopic lipid and the metabolic syndrome. J. Investig. Med..

[36] Garcés-Rimón M., González C., Uranga J.A., López-Miranda V., López-Fandiño R., Miguel M. (2016). Pepsin egg white hydrolysate ameliorates obesity-related oxidative stress, inflammation and steatosis in Zucker Fatty Rats. PLoS ONE.

[37] Aslam M., Madhu S.V. (2017). Development of metabolic syndrome in high-sucrose diet fed rats is not associated with decrease in adiponectin levels. Endocrine.

[38] Singh P., Sharma P., Sahakyan K.R., Davison D.E., Sert-Kuniyoshi F.H., Romero-Corral A., Swain J.M., Jensen M.D., Lopez-Jimenez F., Kara T. (2016). Differential effects of leptin on adiponectin expression with weight gain versus obesity. Int. J. Obes..

[39] Tsuchida A., Yamauchi T., Ito Y., Hada Y., Maki T., Takekawa S., Kamon J., Kobayashi M., Suzuki S., Hara K. (2004). Insulin/Foxo1 pathway regulates expression levels of adiponectin receptors and adiponectin sensitivity. J. Biol. Chem..

[40] Rippe J.M., Angelopoulos T.J. (2016). Relationship between added sugars consumption and chronic disease risk factors: Current understanding. Nutrients.

