# Supplementary material for: High Fat/High Glucose Diet Induces Metabolic Syndrome in an Experimental Rat Model
Source: Nutrients. 2018 Oct 14;10(10):1502. doi: 10.3390/nu10101502 (PMC6213024; doi:10.3390/nu10101502)
Supplement: Supplementary file 1 [file nutrients-10-01502-s001.pdf]

Table S1: Primers used for RT-qPCR in the study.

Gene	Full name	5'→3' primer sequence
18S	18S ribosomal RNA	F: cgccgctagaggtgaaattct
		R: cattcttggcaaatgctttcg
FAS	Fatty Acid Synthase	F: ggccacctcagtcctgttat
		R: agggtccagctagagggtaca
aP2	Adipocyte Protein 2	F: aatgtgcgacgcctttgt
		R: tgatgatcaagttgggcttg
PPARα	Peroxisome proliferator activated receptor α	F: tgcggactaccagtacttaggg
		R: gctggagagagggtgtctgt
Mcad	Medium-Chain Acyl-CoA Dehydrogenase	F: cttagcttctgccctgtggt
		R: tgagagacacttctcaggacctt
Lcad	Long-Chain Acyl-CoA Dehydrogenase	F: gcagttacttgggaagagcaa
		R: ggcatgacaatatctgaatgga
Cpt1β	Carnitine palmitoyltransferase 1β	F: ctcctttcctggacgaggt
		R: gatctggaactgggggatct
Pdk4	Pyruvate dehydrogenase kinase 4	F: gagctgttctcccgctacag
		R: agttctctcacaggcattttctg
PGC1α	PPARγ Coactivator 1α	F: aaagggccaagcagagaga
		R: gtaaatcacacggcgctctt
PGC1β	PPARγ Coactivator 1β	F: ttgacagtggagctttgtgg
		R: gggcttatatggaggtgtgg
Tfam	Mitochondrial transcription factor A	F: agctaaacacccagatgcaaa
		R: tcagctttaaaatccgcttca
Mfn2	Mitofusin-2	F: tcctgggccctaagaatagc
		R: gagaggacgctgaacctgat
Nrf1	Nuclear respiratory factor 1	F: atagtcctgtctggggaaacc
		R: tccatgcatgaactccatct
Tfb2m	Mitochondrial Transcription Factor B2	F: caaaacccatcccatcaact
		R: tcagctttaaaatccgcttca
Opa1	Mitochondrial dynamin-like GTPase	F: ggatttcttcactgcgggta
		R: cggatccatgatctgttgc
Leptin	Leptin	F: ccaggatcaatgacatttcaca
		R: aatgaagtccaaaccggtga
LeptR	Leptin receptor	F: tgtcagaaattctatgtggttttgt
		R: ttggataggccaggttaagtg
Adiponectin	Adiponectin	F: tggtcacaatgggataccg
		R: cccttaggaccaagaacacct
InsR	Insulin Receptor	F: cagaaaaacctcttcaggcaat
		R: ttcaagggatcttcgctttc
Cox1	Cytochrome C oxidase 1	F: tcggaaccctctacctattatttg
		R: ctcgaattagaatacttaaagctgtcc
CycloA	Cyclophilin A	F: ccccatctgctcgcaata
		R: tttgcaatcctgctagacttga
